# Integrated Analysis of lncRNA-Associated ceRNA Network Identifies Two lncRNA Signatures as a Prognostic Biomarker in Gastric Cancer

**DOI:** 10.1155/2021/8886897

**Published:** 2021-09-20

**Authors:** Shuyan Zhang, Shanshan Li, Jian-Lin Guo, Ningyi Li, Cai-Ning Zhang, Jie Liu

**Affiliations:** ^1^Department of Medical Technology Support, Jingdong Medical District of Chinese PLA General Hospital, Beijing, China; ^2^Department of Laboratory, The Seventh Medical Center of Chinese PLA General Hospital, Beijing, China; ^3^Department of Laboratory, Second People's Hospital, Kashgar Area, Xinjiang Province 844000, China; ^4^Department of Laboratory, Guangdong Maoming Agricultural Reclamation Hospital, Maoming, Guangdong Province 525200, China

## Abstract

**Background:**

Gastric cancer (GC) is a malignant tumour that originates in the gastric mucosal epithelium and is associated with high mortality rates worldwide. Long noncoding RNAs (lncRNAs) have been identified to play an important role in the development of various tumours, including GC. Yet, lncRNA biomarkers in a competing endogenous RNA network (ceRNA network) that are used to predict survival prognosis remain lacking. The aim of this study was to construct a ceRNA network and identify the lncRNA signature as prognostic factors for survival prediction.

**Methods:**

The lncRNAs with overall survival significance were used to construct the ceRNA network. Function enrichment, protein-protein interaction, and cluster analysis were performed for dysregulated mRNAs. Multivariate Cox proportional hazards regression was performed to screen the potential prognostic lncRNAs. RT-qPCR was used to measure the relative expression levels of lncRNAs in cell lines. CCK8 assay was used to assess the proliferation of GC cells transfected with sh-lncRNAs.

**Results:**

Differentially expressed genes were identified including 585 lncRNAs, 144 miRNAs, and 2794 mRNAs. The ceRNA network was constructed using 35 DElncRNAs associated with overall survival of GC patients. Functional analysis revealed that these dysregulated mRNAs were enriched in cancer-related pathways, including TGF-beta, Rap 1, calcium, and the cGMP-PKG signalling pathway. A multivariate Cox regression analysis and cumulative risk score suggested that two of those lncRNAs (LINC01644 and LINC01697) had significant prognostic value. Furthermore, the results indicate that LINC01644 and LINC01697 were upregulated in GC cells. Knockdown of LINC01644 or LINC01697 suppressed the proliferation of GC cells.

**Conclusions:**

The authors identified 2-lncRNA signature in ceRNA regulatory network as prognostic biomarkers for the prediction of GC patient survival and revealed that silencing LINC01644 or LINC01697 inhibited the proliferation of GC cells.

## 1. Introduction

Gastric cancer (GC) is a malignant tumour that originates in the gastric mucosal epithelium and has a morbidity and mortality rate ranked second [1, 2]. The incidence of gastric cancer is mainly concentrated in China, Japan, and South Korea. China accounts for 42% of the world's new cases of gastric cancer, and the mortality rate is over 67%. Clinically, most early gastric cancer patients have no obvious symptoms, and a few have nausea, vomiting, or upper gastrointestinal symptoms similar to ulcers, which are difficult to attract enough attention [3]. Previous studies have demonstrated that GC diagnosis and prognosis was evaluated on the basis of disease stage and histological grade [4]. But there were limited predictive values to detect GC using the methods of clinical and pathological symptoms. The development of biological indicators of prognosis are crucial for individualised and precise treatment of GC patients.

Bioinformatics analysis is an important method to investigate the molecular mechanisms of the pathogenesis of tumours and to identify the biological indicators of prognosis according to high-throughput sequencing [5]. Long noncoding RNA (lncRNA) is a class of noncoding RNA without significant protein-coding capacity consisting of 200 nucleotides to 100 kb in length [6]. LncRNAs regulate the expression of target genes transcriptionally and posttranscriptionally and play an important role in the development of cancers [7, 8]. For instance, lncRNA PTEN pseudogene-1 (PTENP1) inhibits cell growth and results in an accumulation of tumour suppressor gene PTEN by adsorbing miR-19 and miR-20a in prostate cancer [9]. In addition, the competing endogenous RNA (ceRNA) hypothesis is proposed as a novel regulatory network, including lncRNAs, microRNAs (miRNAs), mRNAs, and other types of RNAs [10]. Several published studies showed that lncRNAs has the effect of sponging miRNA which weakens the impact of miRNA on mRNA according to the ceRNA hypothesis [11]. In metastatic liver cancer, lncRNA ATB acts as a sponge of the miR200 family to promote cell invasion and deterioration [12]. In breast cancer, lncRNA-GAS5 binds to miR-21 and inhibits the development of breast cancer cells [13]. In gastric cancer, lncRNAs were reportedly involved in many cellular processes including the regulation of cell proliferation [14], cell death [15], tumour angiogenesis [16], invasion, and metastasis of tumour cells [17].

Several studies have identified lncRNAs signatures for the prediction of overall survival based on the ceRNA network. In breast cancer, the 4-lncRNA signature was used to predict overall survival in the lncRNA-related ceRNA network [18]. In melanoma, a 7-lncRNA prognostic signature was established using comprehensive analysis of ceRNA network [19]. In pancreatic cancer, a 7-lncRNA signature was carried out as diagnostic and prognostic biomarkers through ceRNA mechanism [20]. In ovarian cancer, a ten-lncRNA signature was developed as a risk factor in ceRNA network which is involved in stage progression of ovarian cancer [21]. Yet, lncRNA signature for a risk score model based on the ceRNA network for GC patients is rare.

The present study retrieved the expression profiles of lncRNA, miRNA, and mRNA between GC tumour tissue and nontumour tissue from The Cancer Genome Atlas (TCGA) database. The lncRNA-miRNA-mRNA ceRNA network was constructed using integrated analysis. Afterward, functional enrichment analysis were performed to explore the biological roles of lncRNAs in GC. The prognostic value was evaluated by using the Kaplan-Meier method and Cox proportional hazards analysis. Furthermore, we identified novel lncRNAs for the prediction of overall survival and elucidate lncRNA-mediated ceRNA regulatory mechanisms in the prognosis of GC. Finally, our results found that silencing LINC01644 and LINC01697 inhibited the proliferation of GC cells, suggesting that LINC01644 and LINC01697 contribute to the pathogenesis and progression of GC as therapeutic targeting.

## 2. Materials and Methods

### 2.1. Data Resources

RNA sequencing (RNA-Seq) data was derived from The Cancer Genome Atlas (TCGA, https://cancergenome.nih.gov/,version 21.0, release time: December 10, 2019) data portal. A total of 407 individuals with GC were included in this study. A total of 375 tumour tissues and 32 nontumour tissues with available mRNA sequencing and lncRNA sequencing and miRNA data of 436 GC tumour tissues and 41 nontumour tissues were downloaded. Furthermore, GSE65801 (32 gastric tumour tissues and 32 paired adjacent normal tissues) and GSE84787 (10 gastric tumour tissues and 10 paired adjacent normal tissues) databases from the Gene Expression Omnibus (GEO) were downloaded and integrated to reduce the batch effect by sva package in R software as testing data. In addition, the clinical information of GC patients was also downloaded from TCGA and International Cancer Genome Consortium (ICGC) database. The exclusion criteria were that the histological diagnosis was not GC, and there was not enough information for clinical characteristics including age, gender, race, survival status, and survival time. The clinical characteristics for GC patients are listed in Table 1.

### 2.2. Differentially Expression Analysis

Differentially expressed genes were identified by using edgeR package in R including lncRNAs (DElncRNAs), miRNAs (DEmiRNAs), and mRNAs (DEmRNAs). The cut-off criteria were set as a false discovery rate (FDR) less than 0.01 and ∣LogFC | >1.5.

### 2.3. Construction of the ceRNA Network

The lncRNA-miRNA-mRNA network was constructed using Cytoscape 3.7.2 based on the ceRNA hypothesis. The lncRNA-miRNA interaction was predicted using the miRcode database [22], and the miRNA-mRNA interaction was performed using Targetscan, miRTarBase, miRwalk, RAID, and miRDB databases [23]. In addition, the GC-specific RNAs with FDR < 0.01 and ∣LogFC | >2 were reserved including lncRNAs, miRNAs, and mRNAs. Finally, the theory that miRNAs are negatively regulated by lncRNAs and mRNAs was used to establish the ceRNA network [18].

### 2.4. Functional Enrichment Analysis

The function of lncRNAs corresponded to that of the associated mRNAs [18]. To assess the putative biological role of DElncRNAs, functional enrichment analysis was performed using the DEmRNAs in the ceRNA network. Kyoto Encyclopedia of Genes and Genomes (KEGG) pathway enrichments were accomplished using KOBAS (http://kobas.cbi.pku.edu.cn/kobas3) [24]. Gene Oncology (GO) function analyses were performed using “clusterProfiler” package in R, including biological processes (BP), molecular function (MF), and cellular component (CC) [25].

### 2.5. PPI Network Construction and Cluster Identification

PPI network was characterised using the Search Tool for the Retrieval of Interacting Genes (STRING) database (https://string-db.org/cgi/). To investigate the feature and structure of the network, a Cytoscape plug-in called “ClusterONE” was applied to discover densely connected and possibly overlapping regions within the network [26]. The minimum size was set to >30 as the cut-off criterion.

### 2.6. Survival Analysis and Identification of GC-Specific Prognostic Signatures

The Kaplan-Meier and log-rank test methods were used to calculate the overall survival (OS) rate and depict survival curves. In addition, univariate Cox proportional hazards regression analysis was implemented to analyse the relationship between DElncRNAs and OS using the survival package in R. The significance correlation between DEmRNAs and OS was evaluated for GC patients. To determine the prognostic value in patients with GC, multivariate Cox hazards regression model was estimated to determine the independently prognostic factors. All the patients were divided into low-risk and high-risk groups according to the median risk score, which was calculated as follows: Risk score = exp_lncRNA1_∗*β*_lncRNA1_ + exp_lncRNA2_∗*β*_lncRNA2_ + ⋯exp_lncRNAn_∗*β*_lncRNAn_ (“exp” denotes the expression of lncRNA and “*β*” is the regression coefficient of the multivariate Cox regression model) [22]. After that, Kaplan-Meier survival analysis with the log-rank test and receiver-operating characteristic (ROC) curve was established to measure the risk prediction rate of DElncRNAs and assess the results of the risk scoring system. The area under the receiver-operating curve (AUC) was used to indicate the prediction performance. All survival analyses were performed using “survival” package in R software. In addition, the RNA-seq data and clinical data were downloaded from the International Cancer Genome Consortium (ICGC) portal to validate the results as a separate validation cohort.

### 2.7. Cell Culture

The human GC-related cell lines (HGC-27, BGC-823, and SGC-7901) and human normal gastric epithelial cells GES-1 were purchased from American Type Culture Collection (ATCC) Bank. All the cell lines were cultured in RPMI 1640 medium (Sigma-Aldrich, USA) containing 10% foetal bovine serum (FBS) (Gibco, Thermo Fisher Scientific, CA, USA). All these cell lines were incubated in a humidified incubator at 37°C under a 95% air and 5% CO2 condition.

### 2.8. RNA Extraction and Real-Time Quantitative PCR (RT-qPCR)

Total RNA was extracted from cell samples according to the instruction manual of the Trizol reagent (TaKaRa Bio, Dalian, China). Reverse transcription of RNA was performed using Reverse Transcription System (TaKaRa Bio, Dalian, China). The RT-qPCR assays were performed using SYBR® Green Master Mix Kit (TaKaRa Bio, Dalian, China) following manufacturer's protocol. The primer sequences of LINC01644 (NR_109967) and LINC01697 (NR_126010) were as follows: LINC01644-F: CACGCCCTTCCCCTGCACTG; LINC01644-R: CAACAAGGGATGGGATGGGA; LINC01697-F: CCACACACGCGCACACACGA; and LINC01697-R: TGCCTGCTTCATTCTAGCCA, respectively. In addition, GAPDH was used to normalise the expression and the primer sequences as follows: primer-F: 5′-GGAGTCCACTGGTGTCTTCA-3′; primer-R: 5′-GGGAACTGAGCAATTGGTGG-3′. The RT-qPCR program was 5 min at 95°C followed by 40 cycles of 30 s at 95°C and 45 s at 65°C. The results were analysed using the 2^−ΔΔCT^ method [27].

### 2.9. RNA Interference

The target lncRNAs were subcloned into the plasmid pLenti6/V5. The recombinant lentivirus was generated by Shanghai GenePharma Co., Ltd. (Shanghai, China). The negative control (sh-NC) was constructed using SGC-7901 cells that were transfected with an empty vector. The unmanipulated SGC-7901 cells were used as blank control (BC) group.

### 2.10. CCK8 Assay

The SGC-7901 cells transfected with sh-lncRNAs were seeded into the 96-well plates (5 × 10^4^ cells/well). Each well containing DMEM with 10% FBS was cultured at 3 days. After that, 50 *μ*l (10%) Cell Counting Kit 8 (CCK8) (Dojindo Laboratories, Kumamoto, Japan) solution. The plate was incubated for 1.5 h at 37°C. Optical density (OD) values were measured at 450 nm using Thermo Scientific Microplate Reader.

### 2.11. Statistical Analysis

All of the experiments were performed in triplicate, and the data were presented as means ± standard deviation (SD). Student's *t*-test was used to establish statistically significant differences between two groups. The one-way ANOVA was used to assess data from more than two groups. *P* value < 0.05 was considered to be a significant difference. All statistical analyses were performed using SPSS v23.0 and R software.

## 3. Results

### 3.1. Differentially Expressed lncRNA, miRNA, and mRNA

**Figure 1:**
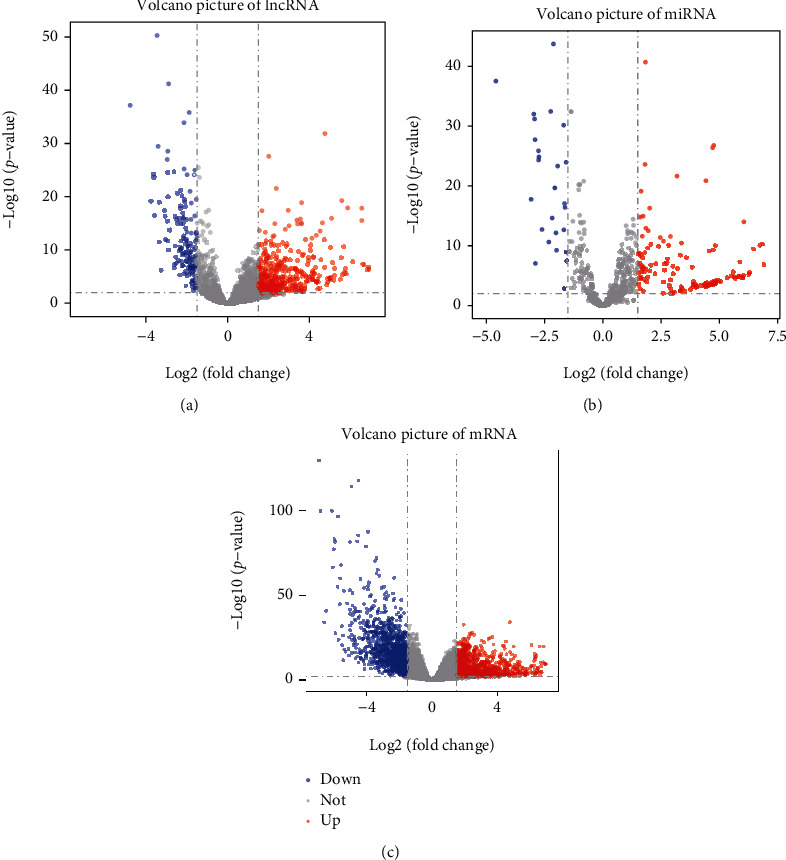
Volcano plots of differentially expressed genes in gastric cancer (GC). (a) Differentially expressed lncRNAs (DElncRNAs). (b) Differentially expressed miRNA (DEmiRNAs). (c) Differentially expressed mRNAs (DEmRNAs). The red dots indicate upregulated genes with FDR < 0.01 and LogFC > 1.5; the blue dots show downregulated genes with FDR < 0.01 and LogFC < −1.5; the grey dots represent genes with no significant difference. FDR: false discovery rate; LogFC: log fold change.

The differential expression of lncRNA, miRNA, and mRNA was explored using TCGA database with FDR < 0.01 and ∣LogFC | >1.5 as the thresholds. A total of 585 DElncRNAs (445 upregulated and 140 downregulated, Figure 1(a)), 144 DEmiRNAs (118 upregulated and 26 downregulated, Figure 1(b)), and 2794 DEmRNAs (1425 upregulated and 1369 downregulated, Figure 1(c)) were identified between GC nontumour tissue and tumour tissue.

### 3.2. Kaplan-Meier Survival Curve Analysis of DElncRNAs and Construction of ceRNA Network

**Figure 2:**
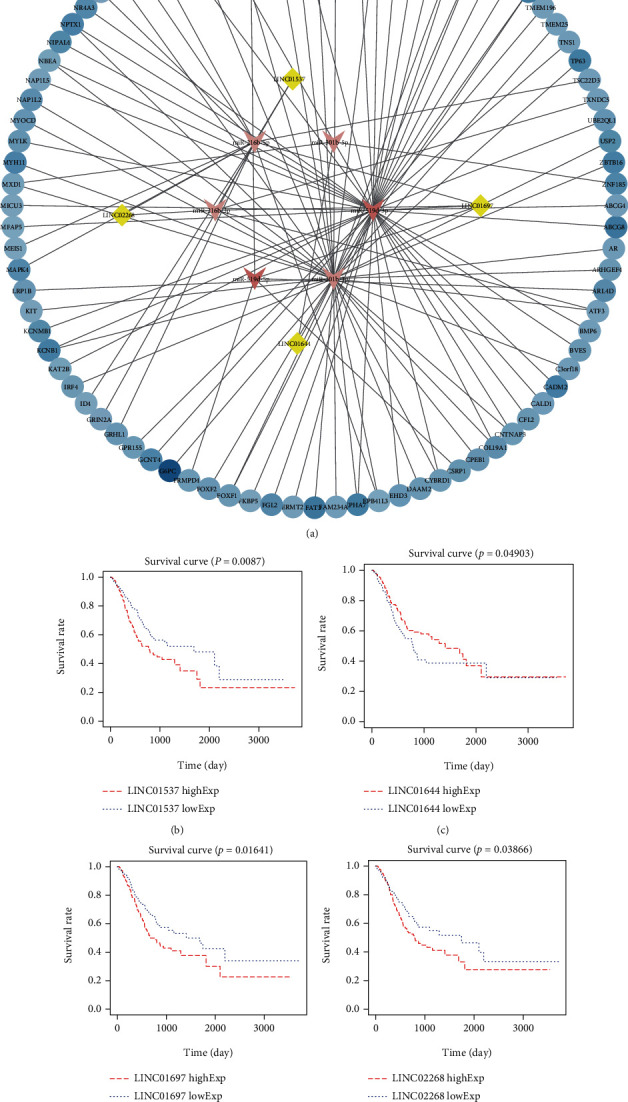
Competing endogenous RNA (ceRNA) network and Kaplan-Meier analysis results of DElncRNAs. (a) The ceRNA network of lncRNA–miRNA–mRNA in GC. Yellow diamonds indicate lncRNA; pink triangles indicate miRNA; blue circles indicate mRNA. (b) Kaplan-Meier survival plot and boxplot of DElncRNAs in ceRNA network including LINC01537, LINC01644, LINC01697, and LINC02268. Log-rank test was used to assess the survival differences and between two groups.

To further verify the prognostic value of DElncRNAs in GC patients, the relationship between DElncRNAs and OS was determined using the Kaplan-Meier method with the log-rank test. The result indicates that 35 were significantly associated with survival (Table 2). Subsequently, the ceRNA network was constructed to investigate the function by which lnRNA regulates the mRNA through sponging miRNA. A total of 35 DElncRNAs were predicted to interact with DEmiRNAs using the miRcode tool. The miRDB, TargetScan, and miRanda programs were used to predict the target mRNA. The 88 target DEmRNAs involved in 2794 DEmRNAs were enrolled in the ceRNA network. Finally, we identified four coexpression DElncRNAs, three coexpression DEmiRNAs, and 88 coexpression DEmRNAs which were selected to establish the ceRNA network (Figure 2(a)). The Kaplan-Meier survival curves of four coexpression DElncRNAs are presented in Figure 2(b), and detailed information about the ceRNA network is shown in Table S1.

### 3.3. Functional Enrichment Analysis

**Figure 3:**
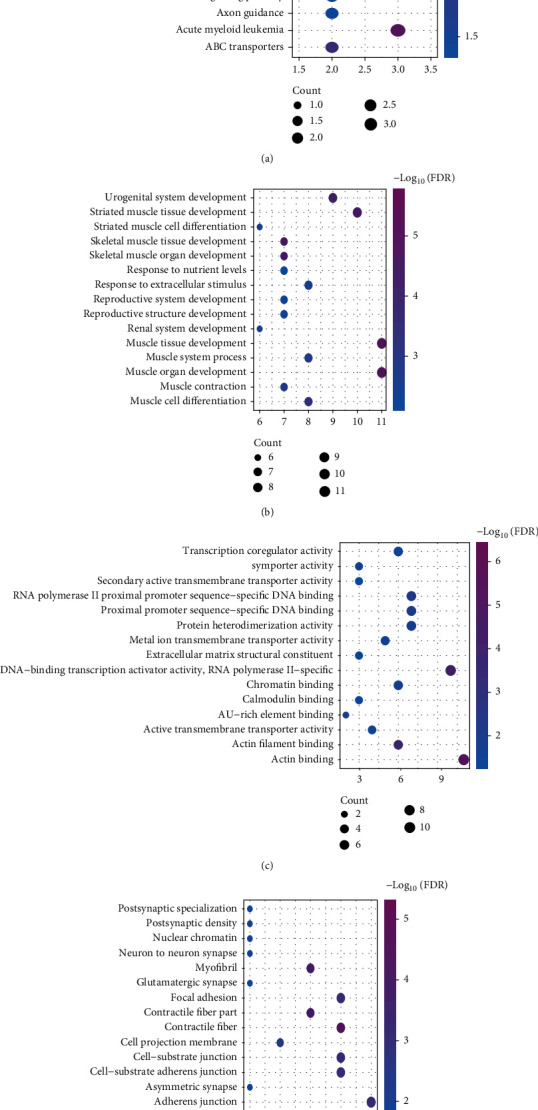
Functional enrichment analysis of DEmRNAs in the ceRNA network. (a) Fifteen most enriched KEGG pathways. Fifteen most enriched GO annotations that consist of three structured ontologies describing biological process (b), molecular function (c), and cellular component (d).

The KEGG pathway and function enrichment of 88 DEmRNAs in ceRNA network were performed using KOBAS 2.0 and clusterProfiler package in R. Results of the KEGG pathway demonstrate that the DEmRNAs were significantly enriched in cancer-related signalling pathways, including TGF-beta, Rap 1, calcium, and cGMP-PKG signalling pathways (Figure 3(a)). Part of the KEGG analytical results are shown in Table 3. Following this, Gene Ontology (GO) analysis revealed 180 biological process (BP) terms, 18 molecular function (MF) terms, and 19 cellular component (CC) terms with *P* value < 0.01 as the cut-off criteria. The top 15 terms in BP involved in muscle tissue development and response to nutrient levels are visualised in Figure 3(b). The top 15 terms in MF and CC were mainly associated with DNA replication, cell division, cell adhesion, and the cellular protein metabolic process (Figures 3(c) and 3(d)).

### 3.4. Protein-Protein Interaction, Prognostic Significance of Hub Genes, and Subnetwork Construction

**Figure 4:**
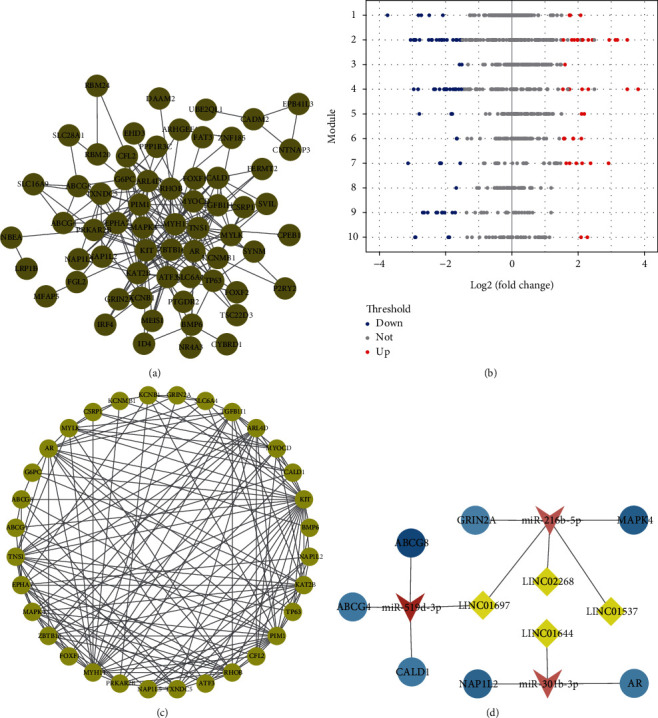
Construction of protein-protein interaction (PPI) network and ceRNA subnetworks. (a) The gastric cancer PPI network was identified for 69/88 DEmRNAs in the ceRNA network. (b) The modules were obtained from the PPI network following ClusterOne algorithm containing 10 modules. Red and blue dots represent up- and downregulated genes, and orange represents mRNAs with no significant difference in expression in the ceRNA network. (c) Interaction network of 32 hub genes. (d) Screening of lncRNA-miRNA-hub gene subnetwork. All shapes in red and blue represent upregulation and downregulation. Yellow diamonds indicate lncRNA; pink triangles indicate miRNA; and blue circles indicate mRNA.

Furthermore, 88 DEmRNAs were sent for construction of protein-protein interaction (PPI) network by the STRING database (Figure 4(a)). Subsequently, the PPI modules were explored with the tools of ClusterONE APP of the Cytoscape (version 1.0, http://www.paccanarolab.org/clusterone) [28]. The results show that 10 PPI modules were identified with *P* values of less than 0.05 as the cut-off threshold (Figure 4(b)). Detailed information including the internal weight and cluster of these genes can be found in Supplementary Table 2. Moreover, GO enrichment analyses were performed for the modules (as listed in Table 4). The results indicate that the differentially expressed genes of modules 3, 9, and 10 involved in basic biological processes including response to insulin, muscle contraction, and metabolic process, and the dysregulated genes of modules 2 and 5 contributed to regulation of the response to the chemical drug.

The nodes with a high degree are considered as important genes in the network and are named “hub genes” [29]. In this study, 32 hub genes were selected in the PPI network with degree > 4 as the cut-off criteria (Figure 4(c)). In addition, to examine the correlation between hub genes and long-term allograft survival, the following seven hub genes were found to be associated with the prognosis of GC patients using Kaplan Meier survival analysis and log-rank test: AR (*P* = 0.0097); MAPK4 (*P* = 0.0357); CALD1 (*P* = 0.0066); ABCG8 (*P* = 0.0211); ABCG4 (*P* = 0.0295); NAP1L2 (*P* = 0.0412); and GRIN2A (*P* = 0.0428). Furthermore, a lncRNA-miRNA-hub gene subnetwork was construction according to the seven hub genes associated with the prognosis of GC patients (as described in Figure 4(d)).

### 3.5. Establishment of the 2-lncRNA Prognostic Model

**Figure 5:**
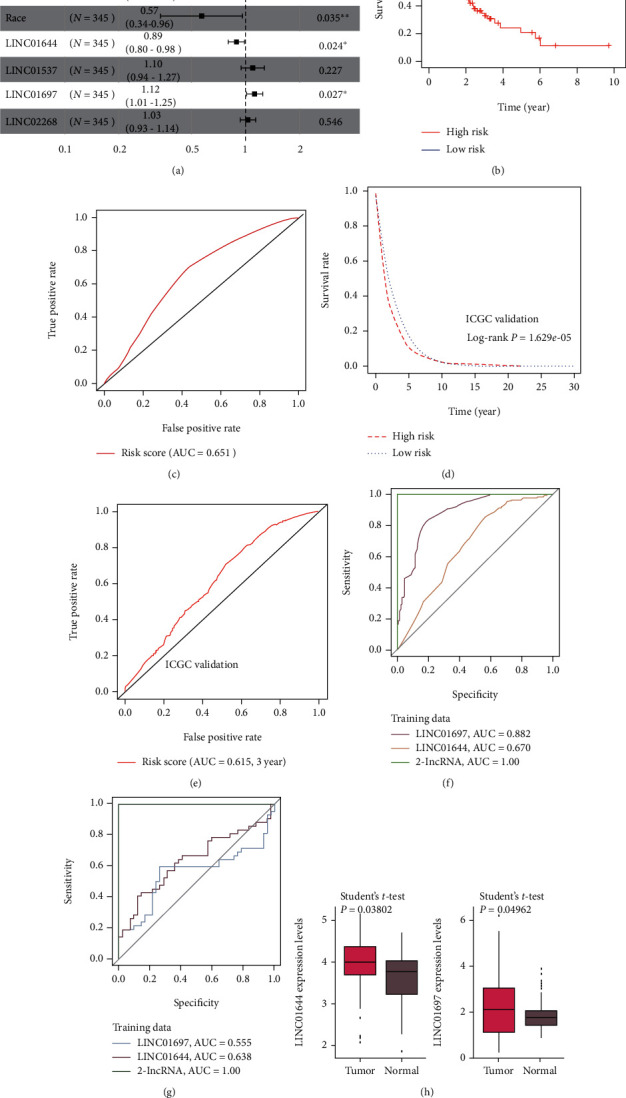
Identification and performance evaluation of the 2-lncRNA signature. (a) Forest plot shows the hazard ratio (HR) and *P* value for overall survival with clinical information and differentially expressed lncRNAs. (b) The survival differences between the high-risk and low-risk groups in TCGA training set. (c) Time-dependent receiver-operating characteristic curve analysis evaluating predictive accuracy of the 2-lncRNA signature for 3-year overall survival in TCGA training set. (d) Kaplan-Meier curves in the high- and low-risk group in ICGC testing cohort. (e) Time-dependent receiver-operating characteristic curve analysis evaluating the predictive accuracy of the 2-lncRNA signature for 3-year overall survival in the ICGC testing cohort. (f) The AUC values of 2-lncRNA compared with single biomarker in TCGA training set. (g) The AUC values of 2-lncRNA compared with single biomarker in GEO testing set integrated with GSE65801 and GSE84787 datasets for reduction of batch effect (g). (h) The expression levels of LINC01644 and LINC01697 were validated using the adjusted GSE65801 and GSE84787 databases to remove the batch effect.

To screen prognostic biomarkers of lncRNAs for GC based on a ceRNA network, a Pearson's chi-square test was performed to identify predictors of a favourable outcome. The results showed significant differences between OS and DElncRNAs in the ceRNA network, including LINC01644 (Chisq = 141, *P* < 0.01), LINC01697 (Chisq = 397, *P* < 0.01), LINC02268 (Chisq = 183, *P* < 0.01), and LINC01537 (Chisq = 161, *P* < 0.01). Furthermore, the prognostic power of four DElncRNAs was assessed using multivariable Cox regression models. All four DElncRNAs involved in the ceRNA network and clinical features including age, gender, and race fitted into the multivariate regression models to detect the significant prognostic value. The results indicate that LINC01644 (*P* = 0.0264), LINC01697 (*P* = 0.0150), age (*P* = 0.0073), and race (*P* = 0.035) were independent influencing factors of survival time (Figure 5(a)). The coefficients in Cox regression of LINC01644 was negative. In contrast, the coefficients in Cox regression of age and LINC01697 were positive. In addition, the DElncRNA expression-based survival risk was calculated. The median risk score was 1.033203. All the patients were divided into low-risk and high-risk groups according to the median risk score. The results show that risk was dramatically correlated with OS, and patients with high-risk scores had a higher mortality rate (Figure 5(b)). Moreover, the signature of DElncRNAs was estimated using the area under ROC curve (AUC) of the ROC curve. The results show that the AUC value of 2-lncRNA signatures in the training set was 0.651, suggesting its utility as a prognostic model for predicting the survival status of GC (Figure 5(c)). To validate the model constructed from the TCGA cohort, the International Cancer Genome Consortium (ICGC) data was calculated by the median value calculated with the same formula as that from the TCGA database [30]. In the present study, the median value was 1.034695. Likewise, patients with high-risk values showed poor prognosis and died earlier (Figure 5(d), *P* = 1.629*e* − 05). In addition, the AUC value of time-dependent ROC curves was 0.615 at 3 years (Figure 5(e)). Next, to further evaluate the 2-lncRNA signature as a potential diagnostic biomarker for GC, the ROC curve analysis was performed using TCGA training data and GEO testing set integrated with GSE65801 and GSE84787 dataset reducing batch effects (Figures 5(f) and 5(g)). The best AUC for a 2-lncRNA signature comprising the two lncRNAs was 1.0, indicating a significant improvement in comparison to each single marker. To validate the expression of the signature (LINC01644 and LINC01697), the GSE65801 (32 gastric tumour tissues, and 32 paired adjacent normal tissues) and GSE84787 (10 gastric tumour tissues and 10 paired adjacent normal tissues) databases in the GEO were downloaded and normalised by limma package in R software. Furthermore, the sva R package was used to remove the batch effect. The results show that LINC01644 (*P* = 0.03802) and LINC01697 (*P* = 0.04962) were significantly upregulated in gastric tumour tissues compared to the adjacent normal tissues in the testing data (Figure 5(h)), which is consistent with the present findings.

### 3.6. Effects of LINC01644 and LINC01697 on Cell Proliferation of GC

**Figure 6:**
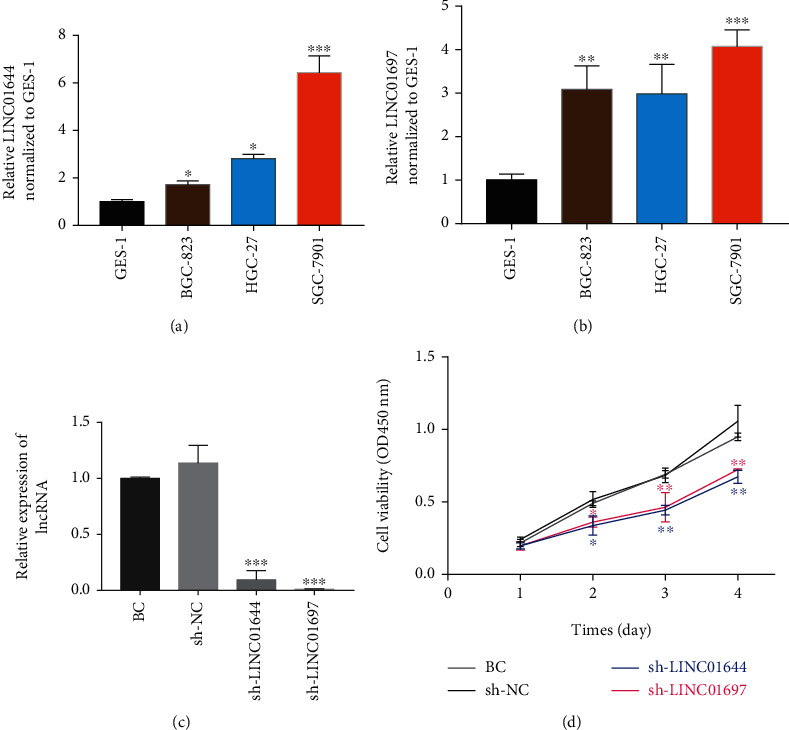
The effect of lncRNAs on the proliferation of gastric cancer cells. (a) The mRNA expression levels of LINC01644 in gastric cancer cell lines and normal GES-1 cells. (b) The mRNA expression levels of LINC01697 in gastric cancer cell lines and normal GES-1 cells. (c) Evaluation of gene silencing of LINC01644 and LINC01697 by transfection of cells with lentiviral shRNAs. (d) CCK-8 assay shows SGC-7901 cell viability after different transfections.

To investigate the expression of LINC01644 and LINC01697, RT-qPCR was used to measure the mRNA expression levels of lncRNAs. Compared to normal GES-1 cells, the relative mRNA expression levels of LINC01644 (Figure 6(a)) and LINC01697 (Figure 6(b)) significantly increased in gastric cancer cells, including SGC-7901, BGC-823, and HGC-27 (*P* < 0.05). To determine the effect of high expression of LINC01644 and LINC01697 on cell proliferation of GC, SGC-7901 cells were infected with shRNA-NC, shRNA-LINC01644, and shRNA-LINC01697. Silencing by shRNA-LINC01644 and shRNA-LINC01644 was confirmed by qRT-PCR. The results show that levels of LINC01644 and LINC01644 were lower in SGC-7901 cells infected with shRNA-LINC01644 shRNA-LINC01644 than that in nontransduced cells or cells infected with shRNA-control, respectively (Figure 6(c), *P* < 0.001). Subsequently, the results of the CCK8 assay show that knockdown of LINC01644 and LINC01697 inhibited tumour cell proliferation of SGC-7901 cells (Figure 6(d)).

## 4. Discussion

Gastric cancer (GC) is one of the most common malignant tumours that seriously endanger human health [31, 32]. In China, gastric cancer has a high morbidity, high metastasis rate, and high mortality [33]. There is thus an urgent need to find useful therapeutic target to reduce the incidence of GC. Recent studies indicate that long noncoding RNA (LncRNA) plays an important role in tumour progression, and ceRNA activity is associated with the development of cancer [34, 35]. In prostate cancer, lncRNA FOXP4-AS1 was identified to promote the growth of cancer cells by sequestering miR-3184-5p to upregulate FOXP4. In GC, silencing lncRNA SPRY4-IT1 suppressed the progression of GC by interacting with miR-101-3p and decreasing inhibiting AMPK expression [36]. Another study established the GC-specific ceRNA network to explore the function according the ceRNA theory. Yet, many studies focus on the function of mRNAs rather than lncRNA. For example, the lncRNA MYOSLID-miR-29c-3p-MCL-1 axis plays a key role in the development of GC, which provides potential new targets for diagnosis and treatment, but identification of the prognostic signature in GC is not recognised [37].

In the present study, a total of 585 differentially expressed lncRNAs (DElncRNAs) were identified between GC tumour tissue and nontumour tissue in the TCGA database. Among the lncRNAs, 35 lncRNAs were identified as significantly associated with overall survival (OS). Studies by Gong et al. indicate a suppressed role of LINC01537 in lung cancer development as a biomarker for survival prediction [38], which is consistent with the associations of LIN01537 expression with OS in GC patients in the present results.

To explore the mechanism of how these lncRNAs expert function, the lncRNA-mediated ceRNA network was constructed based on the ceRNA theory. Four lncRNAs (LINC01644, LINC01537, LINC01697, and LINC02268), six miRNAs, and 88 mRNAs are included in the ceRNA network in GC. Studies show that lncRNA-mediated ceRNA provided novel potential therapeutic targets in colorectal cancer [39]. LncRNA FAL1 was associated with the proliferation and migration of hepatocellular carcinoma cells as a ceRNA mechanism [40]. LncRNA-associated ceRNA contributed to the diagnosis and treatment in squamous cell carcinoma of tongue [41].

The results of the functional enrichment analysis show that nodes in the network significantly participated in the digestive system process including muscle tissue development and in response to nutrient levels and GC-related pathways, such as TGF-beta, Rap 1, calcium, and cGMP-PKG signalling pathways. A previous study showed that the mRNA expression of TGF-beta 1 in gastric cancer might concern the early stage of gastric carcinogenesis [42]. Rap1b expression aberrantly increased in GC, resulting in the inhibition of autophagy and apoptosis of GC cells [43]. Type II cGMP-dependent protein kinase (PKG II) could consequently inhibit the proliferation of GC cells [44]. Taken together, these dysregulated molecules in the network play important roles in the development of gastric cancer.

Numerous studies have shown that lncRNAs plays multitudinous and pivotal roles in the development of cancer as prognostic indicators and potential therapeutic targets [18, 22]. A recent investigation reported that a linear prognostic model of five lncRNAs (C9orf139, MIR600HG, RP5-965G21.4, RP11-436K8.1, and CTC-327F10.4) was considered as prognostic target in pancreatic ductal adenocarcinoma [45]. Seven lncRNAs (AC110491.1, AL451137.1, AC005381.1, AC103563.2, AC007422.2, AC108025.2, and MIR7-3HG) were identified as potential prognostic factors for survival prediction in uterine corpus endometrial carcinoma [46]. A more recent study reported that 4-lncRNA signature independently predicted OS in breast cancer patients [18]. In our study, the relationship between DElncRNAs and OS was determined and two lncRNAs (LINC01644 and LINC01697) showed prognostic value for survival prediction using multivariate cox proportional hazards regression analysis in GC patients. Similarly, previous studies show that a 6-lncRNA signature with prognostic was identified to make the prognosis evaluation of GC patients using robust likelihood-based survival and least absolute shrinkage and selection operator (LASSO) models [47]. Another study identified and validated a 14-lncRNA signature highly associated with the overall survival of patients with GC using C-index, area under the curve, and calibration curves [48]. A total of three lncRNAs (LINC01106, FOXD2-AS1, and AC103702.2) were considered as crucial prognostic factors and showed better accuracy than the TNM pathological staging system in gastric adenocarcinoma [49]. These findings demonstrate that lncRNA signature reveals effective prediction of overall survival in patients with GC.

Subsequently, this study explored the risk scores and found that patients with low-risk had a better survival than high-risk counterparts. These results demonstrate that LINC01644 and LINC01697 are closely related to GC cancer survival. Furthermore, 2-lncRNA signature was an independent prognostic factor in the testing data using multivariate Cox regression. After the literature search, several lncRNA biomarkers in this study have been reported in human diseases. On the contrary, the current investigation showed that LINC01697 was significant protective factor at low expression levels for advanced stages in lung squamous cell carcinoma [50]. Studies have shown that LINC01697 was significantly downregulated as a tumour suppressor in oral squamous cell carcinoma [51]. There are several limitations to the present study. The findings had to be verified using numerous experiment methods, and the molecular mechanism of LINC01697 and LINC01644 in progression and metastasis of gastric cancer will be further investigated.

## 5. Conclusions

In summary, the comprehensive analysis of mRNA, miRNA, and lncRNA expression profiles and clinical features were performed using TCGA, GEO, and ICGC database. Our study identified 2-lncRNA signature as a prognostic factor for survival prediction in GC. Furthermore, silencing LINC01644 and LINC01697 inhibited the proliferation of GC cells. Our results provide novel insights into lncRNA-associated ceRNA network and its roles in the progression of GC.

## Figures and Tables

**Table 1:** The information of clinical features and risk score.

Characteristics	Number of cases	Percentages
Age		
<60	115	33.33
>60	230	66.67
Gender		
Female	125	36.23
Male	220	63.77
Race		
Asian	70	20.29
Black or African American	10	2.90
White	220	63.77
Others	45	13.04
Risk score		
Low	173	50.14
High	172	49.86

**Table 2:** Kaplan-Meier survival analysis for differentially expressed lncRNAs.

LncRNA	*P* value	LncRNA	*P* value	LncRNA	*P* value
HMGA2-AS1	0.0315	LINC01697	0.0164	LINC02268	0.0387
LINC01446	0.0034	LOC339260	0.0067	FLJ42969	0.0195
LINC01644	0.0490	CCDC144NL.AS1	0.0067	TMEM132D-AS1	0.0157
C7orf65	0.0092	CYMP.AS1	0.0187	LOC101928924	0.0331
LINC01537	0.0087	LOC101929532	0.0308	LINC01060	0.0447
LINC01981	0.0253	LINC02042	0.0167	LINC02465	0.0103
CASC20	0.0376	HOXA11.AS	0.0432	LINC02269	0.0212
LOC105373764	0.0476	ADAMTS9-AS1	0.0093	LINC01606	0.0185
ABCA9-AS1	0.0417	LINC02657	0.0402	LINC01592	0.0214
LOC105375787	0.0426	LINC02389	0.0408	LINC01146	0.0451
LINC02182	0.0352	LOC100506388	0.0246	LINC01235	0.0130
LINC02266	0.0199	LOC105369201	0.0252		

**Table 3:** KEGG pathway analysis for differentially expressed genes in ceRNA network (top 15 terms).

KEGG pathway	KEGG entry	Count	Ratio	*P* value
Vascular smooth muscle contraction	hsa04270	4	0.030303	0.000064
Acute myeloid leukaemia	hsa05221	3	0.045455	0.000178
Galactose metabolism	hsa00052	2	0.064516	0.00123
Insulin signalling pathway	hsa04910	3	0.021898	0.001406
ABC transporters	hsa02010	2	0.044444	0.002483
Transcriptional misregulation in cancer	hsa05202	3	0.016129	0.003293
Regulation of actin cytoskeleton	hsa04810	3	0.014019	0.004841
TGF-beta signalling pathway	hsa04350	2	0.021277	0.009971
Insulin resistance	hsa04931	2	0.018519	0.012926
Signalling pathways regulating pluripotency of stem cells	hsa04550	2	0.014286	0.020903
cGMP-PKG signalling pathway	hsa04022	2	0.011976	0.028853
Tight junction	hsa04530	2	0.011765	0.0298
Axon guidance	hsa04360	2	0.01105	0.033378
Calcium signalling pathway	hsa04020	2	0.010363	0.037462
Folate biosynthesis	hsa00790	1	0.038462	0.041275

**Table 4:** The most significant enriched GO terms for the differentially expressed genes involved in 10 modules of PPI network.

Module	Description	Term	Gene number	*P* value
1	Ubiquitin-protein transferase activity	GO:0004842	10	*P* < 0.001
2	Regulation of response to drug	GO:2001023	33	*P* < 0.001
3	Response to insulin	GO:0032868	3	0.002926
4	G protein-coupled receptor activity	GO:0004930	27	*P* < 0.001
5	Response to chemical	GO:0042221	5	0.01508
6	Cell cycle G2/M phase transition	GO:0044839	8	*P* < 0.001
7	Endoplasmic reticulum	GO:0005783	12	*P* < 0.001
8	Vacuole	GO:0005773	1	0.001704
9	Muscle contraction	GO:0006936	10	0.002796
10	Metabolic process	GO:0008152	6	0.001633

## Data Availability

All data generated or analyzed during this study are included in this published article.

## Supplementary Materials

Supplementary 1 Supplementary Table 1: the genes involved in ceRNA network.

Supplementary 2 Supplementary Table 2: the information of modules obtained from the protein–protein interaction network using ClusterOne algorithm.
