# Supplementary material for: Integrated Analysis of lncRNA-Associated ceRNA Network Identifies Two lncRNA Signatures as a Prognostic Biomarker in Gastric Cancer
Source: Dis Markers. 2021 Sep 20;2021:8886897. doi: 10.1155/2021/8886897 (PMC8479203; doi:10.1155/2021/8886897)
Supplement: Supplementary 1 — Supplementary Table 1: the genes involved in ceRNA network. [file 8886897.f1.docx]

Supplementary Table 1 The genes involved in ceRNA network

| Node1 | Node2 | Line |
| --- | --- | --- |
| LINC01697 | mir-519d | lncRNA |
| LINC01644 | mir-301b | lncRNA |
| LINC02268 | mir-216b | lncRNA |
| LINC01697 | mir-216b | lncRNA |
| LINC01537 | mir-216b | lncRNA |
| mir-519d | ABCG4 | mRNA |
| mir-519d | ABCG8 | mRNA |
| mir-301b | AR | mRNA |
| mir-301b | ARHGEF4 | mRNA |
| mir-519d | ARL4D | mRNA |
| mir-216b | ATF3 | mRNA |
| mir-301b | ATF3 | mRNA |
| mir-519d | BMP6 | mRNA |
| mir-519d | BVES | mRNA |
| mir-301b | C3orf18 | mRNA |
| mir-519d | CADM2 | mRNA |
| mir-519d | CALD1 | mRNA |
| mir-519d | CFL2 | mRNA |
| mir-519d | CNTNAP3 | mRNA |
| mir-301b | CNTNAP3 | mRNA |
| mir-519d | COL19A1 | mRNA |
| mir-301b | COL19A1 | mRNA |
| mir-301b | CPEB1 | mRNA |
| mir-519d | CSRP1 | mRNA |
| mir-301b | CYBRD1 | mRNA |
| mir-519d | CYBRD1 | mRNA |
| mir-301b | DAAM2 | mRNA |
| mir-519d | EHD3 | mRNA |
| mir-301b | EPB41L3 | mRNA |
| mir-216b | EPB41L3 | mRNA |
| mir-519d | EPHA7 | mRNA |
| mir-301b | EPHA7 | mRNA |
| mir-301b | FAM234A | mRNA |
| mir-301b | FAT3 | mRNA |
| mir-519d | FERMT2 | mRNA |
| mir-519d | FGL2 | mRNA |
| mir-519d | FKBP5 | mRNA |
| mir-301b | FOXF1 | mRNA |
| mir-519d | FOXF1 | mRNA |
| mir-519d | FOXF2 | mRNA |
| mir-519d | FRMPD4 | mRNA |
| mir-301b | G6PC | mRNA |
| mir-519d | GCNT4 | mRNA |
| mir-519d | GPR155 | mRNA |
| mir-216b | GRHL1 | mRNA |
| mir-216b | GRIN2A | mRNA |
| mir-216b | ID4 | mRNA |
| mir-301b | IRF4 | mRNA |
| mir-519d | KAT2B | mRNA |
| mir-301b | KCNB1 | mRNA |
| mir-519d | KCNB1 | mRNA |
| mir-519d | KCNMB1 | mRNA |
| mir-301b | KIT | mRNA |
| mir-301b | LRP1B | mRNA |
| mir-216b | MAPK4 | mRNA |
| mir-216b | MEIS1 | mRNA |
| mir-519d | MFAP5 | mRNA |
| mir-519d | MICU3 | mRNA |
| mir-301b | MXD1 | mRNA |
| mir-301b | MYH11 | mRNA |
| mir-519d | MYLK | mRNA |
| mir-301b | MYOCD | mRNA |
| mir-301b | NAP1L2 | mRNA |
| mir-519d | NAP1L5 | mRNA |
| mir-301b | NBEA | mRNA |
| mir-519d | NBEA | mRNA |
| mir-519d | NIPAL4 | mRNA |
| mir-519d | NPTX1 | mRNA |
| mir-301b | NPTX1 | mRNA |
| mir-519d | NR4A3 | mRNA |
| mir-216b | P2RY2 | mRNA |
| mir-301b | PCDH10 | mRNA |
| mir-216b | PDZD4 | mRNA |
| mir-216b | PIM1 | mRNA |
| mir-519d | PLCXD3 | mRNA |
| mir-301b | PPP1R3C | mRNA |
| mir-519d | PRKAR2B | mRNA |
| mir-301b | PRUNE2 | mRNA |
| mir-519d | PTGDR2 | mRNA |
| mir-301b | RBM20 | mRNA |
| mir-301b | RBM24 | mRNA |
| mir-519d | RBM24 | mRNA |
| mir-301b | REEP1 | mRNA |
| mir-301b | RHOB | mRNA |
| mir-519d | RNF150 | mRNA |
| mir-301b | SASH1 | mRNA |
| mir-519d | SASH1 | mRNA |
| mir-519d | SLC16A9 | mRNA |
| mir-519d | SLC28A1 | mRNA |
| mir-519d | SLC6A4 | mRNA |
| mir-301b | SVIL | mRNA |
| mir-519d | SVIL | mRNA |
| mir-301b | SYNM | mRNA |
| mir-519d | SYNM | mRNA |
| mir-519d | SYNPO2L | mRNA |
| mir-519d | TGFB1I1 | mRNA |
| mir-301b | TMEM100 | mRNA |
| mir-519d | TMEM100 | mRNA |
| mir-519d | TMEM196 | mRNA |
| mir-301b | TMEM25 | mRNA |
| mir-519d | TNS1 | mRNA |
| mir-301b | TP63 | mRNA |
| mir-216b | TSC22D3 | mRNA |
| mir-216b | TXNDC5 | mRNA |
| mir-519d | UBE2QL1 | mRNA |
| mir-301b | USP2 | mRNA |
| mir-301b | ZBTB16 | mRNA |
| mir-301b | ZNF185 | mRNA |
