# Supplementary material for: Integrated Analysis of lncRNA-Associated ceRNA Network Identifies Two lncRNA Signatures as a Prognostic Biomarker in Gastric Cancer
Source: Dis Markers. 2021 Sep 20;2021:8886897. doi: 10.1155/2021/8886897 (PMC8479203; doi:10.1155/2021/8886897)
Supplement: Supplementary 2 — Supplementary Table 2: the information of modules obtained from the protein–protein interaction network using ClusterOne algorithm. [file 8886897.f2.docx]

**Supplementary Table 2 The information of modules obtained from the protein–protein interaction network using ClusterOne algorithm**

| Modules | Size | Density | Internal weight | External weight | P-value |
| --- | --- | --- | --- | --- | --- |
| 1 | 170 | 0.8446 | 12100 | 1356 | 0.001 |
| 2 | 134 | 0.5089 | 4535 | 1527 | 0.001 |
| 3 | 100 | 0.5008 | 2479 | 2052 | 0.001 |
| 4 | 92 | 0.6278 | 2628 | 1989 | 0.001 |
| 5 | 61 | 0.6661 | 1219 | 415 | 0.001 |
| 6 | 63 | 0.7409 | 1447 | 588 | 0.001 |
| 7 | 36 | 0.9111 | 574 | 52 | 0.001 |
| 8 | 37 | 0.7733 | 515 | 317 | 0.001 |
| 9 | 31 | 0.6108 | 284 | 263 | 5.87E-07 |
| 10 | 42 | 0.6411 | 552 | 744 | 0.000167 |
